# Prediction of the impact of tobacco waste hydrothermal products on compost microbial growth using hyperspectral imaging combined with machine learning

**DOI:** 10.3389/fmicb.2024.1476803

**Published:** 2024-11-05

**Authors:** Dandan Liu, Xinxin Ma, Changwen Ye, Yiying Jin, Kuo Huang, Chenqi Niu, Ge Zhang, Dong Li, Linzhi Ma, Suxiao Li, Guotao Yang

**Affiliations:** ^1^China Tobacco Standardization Research Center, Zhengzhou Tobacco Research Institute, Zhengzhou, China; ^2^School of Environment, Tsinghua University, Beijing, China; ^3^College of Physical Engineering, Zhengzhou University, Zhengzhou, China

**Keywords:** composting, hydrothermal, hyperspectral imaging, machine learning, tobacco waste

## Abstract

The insufficient understanding of the impact of hydrothermal products on the growth characteristics of compost microorganisms presents a significant challenge to the broader implementation of hydrothermal coupled composting for tobacco waste. Traditional biochemical detection methods are labor-intensive and time-consuming, highlighting the need for faster and more accurate alternatives. This study investigated the effects of hydrothermal treatment on tobacco straw products and their influence on compost microorganism growth, using hyperspectral imaging (HSI) technology and machine learning algorithms. Sixty-one tobacco straw samples were analyzed with a hyperspectral camera, and image processing was used to extract average spectra from regions of interest (ROI). Hierarchical cluster analysis (HCA) and principal component analysis (PCA) were applied to assess four key variables: nicotine content, total humic acid content, *Penicillium chrysogenum* H/C ratio, and *Bacillus subtilis* OD_600_ ratio. The effects of hydrothermal treatment on compost were classified as promoting, inhibiting, or neutral regarding microbial growth. The Competitive Adaptive Reweighted Sampling (CARS) method identified the most influential wavelengths in the 900-1700 nm spectral range. The Random Forest (RF) model outperformed SVM, KNN, and XGBoost models in predicting microbial growth responses, achieving *R*_c_ = 0.957, RMSE = 3.584. Key wavelengths were identified at 1096 nm, 1101 nm, 1163 nm, 1335 nm, and 1421 nm. The results indicate that hyperspectral imaging combined with machine learning can accurately predict changes in the chemical composition of tobacco straws and their effects on microbial activity. This method provides an innovative and effective means of improving the resource usage of tobacco straws in composting, enhancing sustainable waste management procedures.

## Highlights

- HCA and PCA sorted hydrothermal treatment impact on composting into three types.
- Four machine learning models were used to predict the most influential wavebands.
- Random Forest model showed superior performance with an R_c_ of 0.957.
- Nicotine content and total humic acid were variables predicting bio-compostability.

## 1 Introduction

China leads the world in tobacco production and consumption (Fang et al., 2020). In 2022, the country generated about 5 million tons of tobacco waste, with straws making up over 60% of this amount (Ma et al., 2023). Traditional disposal methods, such as on-site stacking or centralized incineration, are resource-intensive and cause significant environmental pollution (Zou et al., 2021; Banožić et al., 2020). It is imperative to identify effective methods for the treatment and reutilization of tobacco waste to promote environmental and resource conservation.

Recently, hydrothermal coupling composting technology has emerged as a key method for efficiently and environmentally processing tobacco straws (Yu et al., 2022). Studies demonstrate that hydrothermal treatment can efficiently eliminate detrimental compounds, including heavy metals and pesticide residues, from tobacco straws while enhancing the degradability of cellulose, hemicellulose, and lignin (Sethupathy et al., 2022). However, the complex nature of the products after hydrothermal treatment poses challenges. During the process, nicotine and other inhibitors may be generated or released, potentially inhibiting the activity of compost microorganisms and thereby affecting composting efficiency and product quality (Sun et al., 2022). Therefore, improving the effectiveness of this treatment strategy depends on accurately predicting how these hydrothermal compounds would affect compost microorganisms.

Traditional methods for assessing the impact of hydrothermal products on compost microorganisms include chemical analysis, microbial experiments, and statistical analysis (Gao et al., 2012; Sarker et al., 2021; Mishra et al., 2021). Chemical analysis necessitates substantial reagents and apparatus, while microbial experiments require thorough control of experimental conditions, even though these methods can provide precise evaluations. Their high costs frequently impede the practical application of these methods, as well as extended data acquisition cycles, low prediction accuracy, and challenges in handling complex data. As a result, it is imperative to establish a standardized, efficient, and rapid methodology for assessing the impact of tobacco straw hydrothermal products on compost microorganisms.

Hyperspectral Imaging (HSI) is an advanced technology that combines spectral and imaging capabilities, enabling the rapid and non-destructive acquisition of detailed spectral information from samples (Mahanti et al., 2022). HSI can disclose intricate details about the components within a sample by analyzing spectra at various wavelengths. HSI is particularly well-suited for analyzing complex biomass samples, as it provides quick analysis without the necessity of sample pretreatment, in contrast to traditional chemical and biological analysis methods (Wang et al., 2023). For example, Ahn et al. (2021) accurately determined chlorophyll concentrations in lake water, thereby demonstrating the efficacy of HSI in the early detection of algal blooms. Similarly, Khan et al. (2022) showed that HSI could rapidly collect spectral data from various biomass types without causing any damage to the samples, thereby enabling the precise classification of the biomass based on its spectral characteristics.

However, analyzing HSI data is challenging due to its complexity and high dimensionality, which complicates the extraction of meaningful insights using traditional statistical methods (Ayesha et al., 2020). Machine learning (ML) techniques offer a promising alternative for efficiently and accurately analyzing such complex datasets by automatically identifying patterns and relationships within the data. In hyperspectral imaging, ML algorithms are particularly effective for classification, feature selection, regression, and anomaly detection tasks. ML can develop complex non-linear predictive models by learning the relationships between the spectral data of numerous known samples and their corresponding physicochemical indicators (Ang and Seng, 2021). These models can quickly process large datasets, uncover subtle relationships between spectral data and measured indicators, and provide rapid and accurate predictions for unknown samples.

Numerous studies have successfully combined hyperspectral imaging (HSI) with machine learning (ML) techniques in agriculture, food, medicine, and environmental science, yielding significant results in various applications. Elsherbiny et al. (2021) effectively predicted the water content of the rice canopy by evaluating multivariate techniques and feature selection methods. They applied crop spectral reflectance to various levels of water deficit. The EFS (Ensemble Feature Selection) method, which was validated for wheat yield prediction, was developed by Fei et al. (2022). This method combines deep neural networks (DNN) and hyperspectral vegetation indices, reducing breeding labor and optimizing field management practices. Yang et al. (2021) used hyperspectral data and machine learning to monitor urban black-odorous water. This study used Lasso regression to investigate the synchronized hyperspectral bands and their combinations, using three multivariate regression models to accurately monitor urban black-odorous water bodies.

Although this study offers important references for integrating HSI and ML approaches, a gap persists in predicting biomass processing and its interactions with microbial communities. This research tackles this deficiency by presenting competitive adaptive reweighted sampling (CARS) for feature selection, effectively mitigating multicollinearity challenges in high-dimensional datasets. Furthermore, random forest, SVM, and XGBoost models were employed to attain accurate predictions of microbial growth under limited sample conditions. Table 1 delineates the advancements in relevant research, elucidating the distinctions and contributions of this study in contrast to previous studies.

**Table 1:** Advances in hyperspectral machine learning across different fields.

Research field	Machine learning models	Size	Feature selection methods	Key findings and contributions	References
Agriculture (Irrigation Management)	BPNN, RF, and PLSR	128	VI, MF, and PCA	Predicted plant water content, enabling proactive measures for precision irrigation.	Elsherbiny et al. (2021)
Agriculture (Yield Prediction)	DNN	207	MDI, Boruta, FeaLect, and RReliefF	An integrated feature selection (EFS) method is proposed to predict crop yield accurately.	Fei et al. (2022)
Food (Toxin Detection)	PCNN	2510	FS	Developed a PCNN-FS framework combined with HSI to identify moldy peanuts and predict aflatoxins.	Yuan D. et al. (2022)
Food (Quality Classification)	RF, PLS, and RNN	150	–	Developed a non-destructive prediction model for SSC, titratable acidity, and lycopene in processing tomatoes.	Zhao et al. (2023)
Environmental Monitoring (Water Quality)	SVM, RF, and NN	58	ReliefF and RFE	Effectively identified and monitored urban black-odorous water bodies.	Yang et al. (2021)
Medicine (Disease Classification)	SVM, RF, and CNN	13	–	Provided a promising non-invasive method for brain tumor classification using HSI.	Urbanos et al. (2021)
Biomass (Hydrothermal Treatment of Tobacco Straw)	RF, SVM, and XGBoost	61	CARS	Utilized CARS for efficient feature selection and microbial growth prediction under small sample conditions.	This work

Despite the tremendous advancements in their application across various sectors, there is still an apparent gap in the application of HSI and ML techniques for forecasting microbial interactions with biomass, especially in the hydrothermal treatment of biomass like tobacco straw. Current models do not adequately capture biomass digestion’s intricate biochemical and microbiological dynamics. Previous studies have focused mainly on environmental monitoring, crop management, and food quality, with little emphasis on the complicated interactions between biomass components and microbial communities. By combining HSI and ML approaches to forecast microbial growth in hydrothermally treated biomass, this study fills this research gap. This study presents a novel method for precisely forecasting microbial behavior in limited sample situations by utilizing sophisticated machine learning models, including Random Forest, SVM, and XGBoost, and competitive adaptive reweighted sampling (CARS) for feature selection. The necessity of this study lies in its potential to advance the understanding of biomass processing, enhance resource utilization, and contribute to more efficient bioengineering practices.

Therefore, using hyperspectral imaging (HSI) data, this study created a machine learning model to forecast how hydrothermally treated tobacco straw (HTS) products will affect the growth traits of compost microorganisms. The main objective of this article is to classify HTS products based on their associated microbial growth characteristics by selecting specific wavelengths from HSI scans, focusing on the near-infrared spectrum range (900–1700 nm). A predictive machine learning model was developed utilizing HSI image data of HTS products and their associated microbial growth features. This project seeks to improve the efficiency and precision of feasibility assessments for hydrothermal coupling composting of tobacco straw, minimize waste and expenses, and facilitate the broader use of hydrothermal coupled composting technology.

## 2 Materials and methods

### 2.1 Tobacco straws and composting microorganisms

The tobacco straws utilized in this research were obtained from Xiang County, Xuchang City, Henan Province, a prominent tobacco cultivation region in China. Following harvest, the straws underwent natural air-drying. The chemical properties of tobacco straw can be found in Supplementary Table S1. The selected composting microorganisms for this investigation are *Penicillium chrysogenum* and *Bacillus subtilis* FYZ1-3, isolated from tobacco waste and maintained in our laboratory (Tian et al., 2023; Ye et al., 2023). These strains have shown considerable effectiveness in decomposing tobacco waste during composting and are utilized as representatives of fungi and bacteria in this research. Cultivation occurred in both solid and liquid LB medium.

### 2.2 Hydrothermal treatment of tobacco straws

The hydrothermal treatment was conducted using a WZC-small batch intermittent high-pressure reactor (Wuzhouding Technology Co., Ltd., Beijing, China). This reactor, made of 316 stainless steel, operates safely at pressures up to 22 MPa, with a heating range of 0 to 300°C, and has an effective volume of 500 mL. The apparatus is additionally fitted with a magnetic, mechanical stirring device. Sixty distinct hydrothermal treatment conditions were established to account for hydrothermal temperatures and duration’s combined effects based on the hydrothermal intensity expression (Equation 1) (Chornet and Overend, 1991). The temperature ranged from 0 to 260°C, with treatment durations spanning 20–180 min. A control group, subjected to the same pretreatment without heating, was also included. In total, 61 hydrothermal product samples were generated. Detailed treatment conditions for each group can be found in Supplementary Table S2.


(1) $$\log R_{0}=\log\left[\overset{n}{\underset{i=1}{\sum }}t_{i}\exp\left(\frac{T_{i}-T_{b}}{\omega }\right)\right]$$

Where *logR_0_* is the intensity factor; *t_i_* represents reaction time in minutes; *T_i_* represents reaction temperature in °C; *T_b_* = 100°C is the reference temperature; and *ω* = 14.5 is the fitting parameter.

A high-speed grinder was used to fine-mill the dry tobacco straws after they had been crushed in multiple stages. A 60-mesh screen filtered the resultant material, yielding a homogenous powder with an average particle size of roughly 0.25 mm. A slurry was created for the experiment by combining 30 g of this tobacco straw powder with 300 mL of deionized water at a 1:10 ratio. This mixture was then transferred to the high-temperature–pressure reactor, heated gradually at a rate of 5°C per minute to the set temperature, and maintained for the specified duration. After the treatment, the reactor was rapidly cooled with water depressurized, and the liquid was filtered using a 0.22 μm filter. The hydrolysate was stored at 4°C, with a portion frozen at −80°C for 6 h and then freeze-dried for 24 h to obtain the solid sample.

### 2.3 Detection of physicochemical properties of HTS

The nicotine content of the samples was analyzed using gas chromatography–mass spectrometry (GC–MS) (Hossain and Salehuddin, 2013). A 0.5 g freeze-dried sample was dissolved in 10 mL of isopropanol, shaken for 30 min, and then centrifuged at 5000 rpm for 10 min at 4°C. The filtered supernatant was analyzed using a DB-5MS column (30 m × 0.25 mm, 0.25 μm) with an injection port temperature of 250°C and a split ratio of 10:1. The column temperature was initially set at 80°C for 2 min, then increased at a rate of 10°C/min to 280°C, where it was held for 5 min. Helium was used as the carrier gas at a 1 mL/min flow rate. The mass spectrometer operated at 70 eV with an ion source temperature of 230°C and a quadrupole temperature of 150°C, scanning from m/z 50–550. Nicotine content was determined based on retention time and mass spectra.

The alkali dissolution-acid precipitation method determined the total humic acid (HA + FA) content in the solid powder samples (Lamar and Talbot, 2009). A 1.0 g sample was mixed with 100 mL of 0.1 M NaOH and shaken for 24 h. After filtering the extract and adjusting the pH to 1.0 with 0.1 M HCl, humic acid (HA) was precipitated and separated by centrifugation before being dried and weighed. After filtering and drying the residual solution, the residue was dissolved in 0.1 M NaOH. To precipitate fulvic acid (FA), the pH was once more brought to 1.0. The combined weights of HA and FA indicated the total amount of humic acid. The mean ± standard deviation from three tests was used to present the results.

### 2.4 Impact assessment of HTS on composting microbial growth

While the control group was given sterile water, the experimental group was treated with liquid-phase products from section 2.2 at a 3% concentration in an LB culture medium. The 3% concentration was chosen because prior research showed that it successfully demonstrated the impact of the hydrothermal products on microbial growth without producing undue stimulation or inhibition. Moreover, this concentration is consistent with results from earlier research (Kamal et al., 2022), where 3% was identified as an optimal concentration for assessing the effects of hydrothermal products in composting or fermentation processes. LB solid culture medium was then inoculated with *Penicillium chrysogenum* and placed in a 28°C environment for 48 h. Colony sizes were subsequently measured and compared between the experimental and control groups.

At the same time, 3% *Bacillus subtilis* seed liquid was added to the LB liquid culture medium for both the experimental and control groups. Bacterial growth was evaluated by measuring optical density at 600 nm (OD_600_) and comparing ratios between the experimental and control groups after an overnight shaking incubation at 37°C and 180 rpm. To ensure the precision and repeatability of the findings, every experiment was carried out in triplicate under sterile conditions.

### 2.5 Classification of HTS

Hierarchical cluster analysis (HCA), an unsupervised method for pattern recognition, was applied to group hydrothermally processed tobacco straw products based on their effects on compost microbial growth. These products were categorized according to their physical and chemical properties and their impacts on microbial growth, including factors like nicotine content, total humic acid content, *Penicillium chrysogenum* colony ratio, and bacterial OD_600_ ratio. The data were standardized and analyzed using the Ward method, enabling sample classification according to microbial effects and establishing validation groups essential for machine learning (Xiang et al., 2023). This method successfully classified hydrothermal products into three categories according to their influence on compost microbial growth: those that enhance growth, those that have no significant effect, and those that suppress growth.

### 2.6 Hyperspectral data acquisition

The hyperspectral analysis system, supplied by Shenzhen HyperNano Optical Technology Co., Ltd., utilizes advanced industrial technology featuring a MEMS chip hyperspectral camera. Operating within a wavelength range of 900–1700 nm, it offers a spectral resolution of 5 nm across 125 spectral channels. The system includes a dark chamber with a halogen light source (400–2,500 nm), adjustable from 0 to 12 V to ensure illumination uniformity exceeding 95%.

The hyperspectral imaging system was preheated for 30 min before scanning to ensure stability. The voltage of the light source was established at 9 V, with an exposure duration of 30 ms selected. Hydrothermal products from each experimental group were uniformly distributed on a circular plate and scanned in a dark chamber three times. Each scan incorporated white reference images (using a standard whiteboard with reflectance >99%) and dark reference calibration images. The sample core was placed in a Petri dish during scanning to minimize translation inertia.

To compensate for variations in light intensity and dark current within the sensor, a calibration (Equation 2) was employed to adjust the reflectance of hyperspectral images:


(2) $$R=\frac{I_{0}-I_{B}}{I_{W}-I_{B}}$$

Where *R* represents the calibrated reflectance hyperspectral image, *I_0_* denotes the original hyperspectral image captured, *I_W_* signifies the hyperspectral image of a whiteboard with a reflectance of 99%, used as the white reference, and *I_B_* refers to the hyperspectral image of a dark reference obtained when the lens is covered (Weng et al., 2021).

### 2.7 Spectral reflectance extraction

The average spectral reflectance of tobacco hyperspectral images was derived using a masking approach. Initially, the ratio of all band pairs across the hyperspectral images was computed to identify the maximum band ratio, highlighting the greatest difference in reflectance between the tobacco and its background. A mask was subsequently generated using the maximum band ratio to isolate the tobacco region. A threshold segmentation algorithm was subsequently employed to isolate the tobacco hyperspectral image from its background effectively. The average spectral reflectance of the tobacco hyperspectral image was calculated by averaging the spectral reflectance values of all pixels within the designated tobacco region.

### 2.8 Modeling methods

#### 2.8.1 Data preprocessing and feature selection

Data preprocessing and feature selection are essential for enhancing the performance of machine learning models when dealing with hyperspectral data. This study employed the Savitzky–Golay (SG) filtering method to smooth hyperspectral data, reduce noise, and improve spectral signal quality. The parameters selected for SG filtering included a window width of *w* = 13 and a polynomial order of *p* = 2. This method effectively balances data smoothing with the preservation of spectral details, thereby increasing the accuracy and reliability of subsequent analyses. For feature selection, the Competitive Adaptive Reweighted Sampling (CARS) method was used to identify the most influential spectral bands for modeling. The CARS method iteratively reduces the size of the feature set by adjusting competitive weights, ultimately selecting the most representative and informative bands. The parameters for the CARS method in this study included a sample size of *N* = 50, a stopping factor of *f* = 20, and a cross-validation fold number of cv = 10. The selected parameters aim to enhance feature selection, thereby maximizing the extraction of relevant information from the hyperspectral data. This method effectively decreases model complexity while preserving essential features that improve predictive accuracy and interpretability.

#### 2.8.2 Machine learning models

Machine learning models can discern patterns and relationships within data and are particularly useful for handling complex tasks. This study employed four distinct machine learning regression algorithms: Support Vector Machine (SVM), K-Nearest Neighbors (KNN), Extreme Gradient Boosting (XGBoost), and Random Forest (RF). The parameters for each model were not defaulted but instead optimized through hyperparameter tuning techniques, such as grid search and cross-validation, to achieve optimal performance. We fine-tuned the kernel function (e.g., linear or RBF) and the SVM model’s regularization parameter (C value). In the KNN model, the number of neighbors (K value) and the distance metric were adjusted. For XGBoost, key parameters such as the learning rate, tree depth, and subsample ratio were optimized. Similarly, for the Random Forest model, we optimized the number of trees, maximum tree depth, and minimum sample size required for node splitting. Each algorithm has distinct advantages: SVM is effective for high-dimensional datasets, utilizing kernel functions to manage non-linear problems and demonstrating strong generalization abilities (Joachims, 2012). KNN is conceptually simple, makes no assumptions regarding data distribution, and classifies new instances based on the proximity to the nearest K samples. It is especially efficacious for small datasets with minimal noise (Maillo et al., 2019). XGBoost is an effective gradient-boosting algorithm that systematically constructs weak learners to improve model precision. It manages non-linear interactions and extensive datasets (Kavzoglu and Teke, 2022). RF improves classification or regression accuracy by constructing multiple decision trees and combining their results. It exhibits strong resistance to overfitting and is suitable for high-dimensional datasets or those with missing values (Zhu, 2020). Selecting appropriate models and tuning their parameters is crucial for achieving optimal performance. A thorough analysis of the characteristics and limitations of each model can lead to the best modeling outcomes.

#### 2.8.3 Evaluation metrics

The study evaluated four algorithms using six critical parameters: calibration correlation coefficient (r_c_), prediction correlation coefficient (r_p_), calibration coefficient determination (R_c_), prediction coefficient determination (R_p_), calibration root mean square error (RMSEC), and prediction root mean square error (RMSEP) (Haruna et al., 2022). Following a thorough performance analysis, the top-performing model was identified. These metrics were selected as they provide complementary insights into the model’s accuracy and robustness. The combination of correlation and determination coefficients offers a comprehensive evaluation of the linear relationship and the model’s ability to explain variance in the data. At the same time, RMSE highlights prediction accuracy by quantifying the deviation between predicted and actual values.

Because of the comparatively lower variability in the hydrothermal treatment data, the residual predictive deviation (RPD), which is occasionally employed in domains with significant data variability, such as soil or food quality analysis, was considered less pertinent for this investigation. The measures offer a thorough and reliable assessment of the model’s performance.

Internal validation using K-fold cross-valuation helped improve the model’s generalizing and dependability. This approach randomly divided the dataset into k subsets, utilizing each once as a validation set and training from the remaining k-1 subsets. This process was repeated k times, and the average performance metrics across all iterations were calculated. Cross-validation helped prevent overfitting and provided a reliable estimate of the model’s performance on unseen data.

### 2.9 Statistical analysis

The impact of hydrothermal products on microbial growth was studied through three repetitions, and the data underwent variance analysis using the Tukey test at a significance level of *p* < 0.05. MATLAB 2022a was employed for image processing, and Python 3.8.3 with Jupyter Notebook was utilized for data processing and developing machine learning models.

## 3 Results and discussion

### 3.1 Index content and statistical characteristics for HTS

The results for the 61 hydrothermal product samples, including nicotine content, total humic acid levels, and the growth performance of *Penicillium chrysogenum* and *Bacillus subtilis* under different hydrothermal conditions, were provided in Supplementary Table S2. This included detailed data on each hydrothermal condition and the corresponding characteristics of the resulting products.

A statistical analysis of the experimental dataset was performed to ensure the model’s robustness and accuracy, with the findings in Table 2. The table presents a comprehensive summary of the principal statistical attributes for the calibration and validation datasets, encompassing the mean, standard deviation, minimum, and maximum values. Although the *Bacillus subtilis* OD_600_ ratio exhibits a relatively high standard deviation, common in chemical and biological experiments, the other data do not show significant natural variability.

**Table 2:** Statistical characteristics of calibration and validation data for hydrothermal product indicators.

Indicator	Mean	Std	Min	Max
Nicotine	5.35	5.29	0.13	20.43
HA + FA	58.01	7.07	33.40	68.80
*Penicillium chrysogenum* H/C ratio	92.72	10.80	71.47	111.09
*Bacillus subtilis* OD_600_ ratio	89.09	54.93	6.42	156.69

The statistical characteristics demonstrate the diversity of hydrothermal products under varying settings, indicating that the dataset encompasses a broad spectrum of experimental scenarios, hence facilitating subsequent model calibration and validation. Furthermore, the results demonstrate substantial disparities among the assessed variables, affirming the model’s resilience in managing intricate data.

### 3.2 Classification of HTS

**Figure 1:**
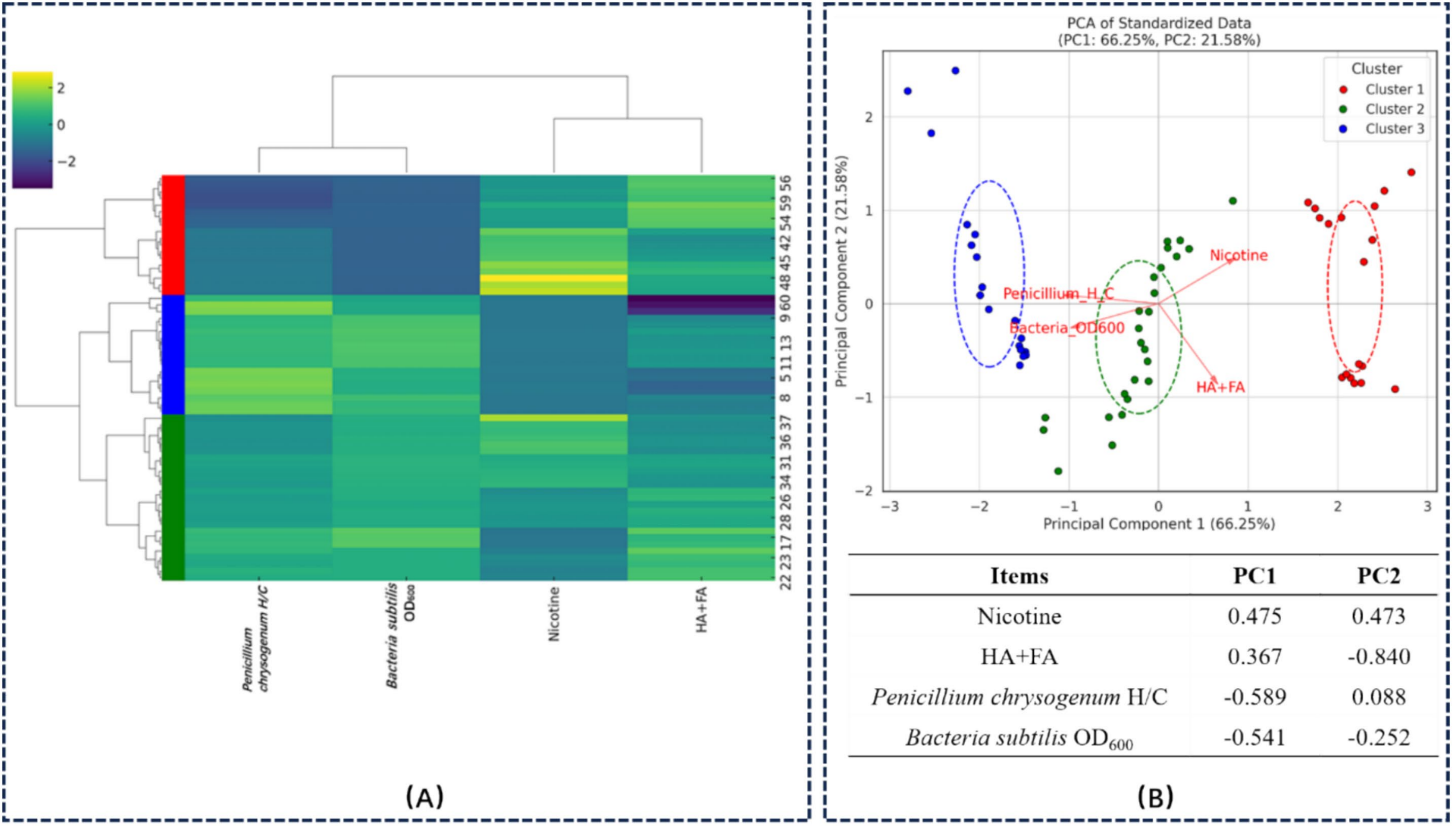
Classification of HTS based on their impact on composting microorganisms using **(A)** HCA and **(B)** PCA with loading scores. Clusters 1, 2, and 3 are represented by red, green, and blue, respectively.

This study employed hierarchical cluster analysis (HCA) and principal component analysis (PCA) to categorize tobacco straw samples subjected to varying hydrothermal intensities into three distinct groups, as depicted in Figure 1. This classification elucidates the distribution characteristics of the samples based on four key variables: nicotine content, total humic acids (HA + FA) content, the ratio of *Penicillium chrysogenum* H/C, and the ratio of *Bacillus subtilis* OD_600_. The results demonstrated how these variables influence the categorization of the treated samples.

Figure 1A displays the hierarchical clustering dendrogram, which visually represents the similarity and clustering process of the data points. The vertical axis denotes the distances between samples, while the horizontal axis illustrates the relationships among samples and clusters. The data is segmented into three primary clusters by choosing a suitable distance threshold, each identified by distinct colors: red for Cluster 1, green for Cluster 2, and blue for Cluster 3.

Figure 1B illustrates the PCA results, where high-dimensional data is projected onto a two-dimensional plane. Principal Component 1 (PC1) accounts for 66.25% of the variance, and Principal Component 2 (PC2) accounts for 21.58%, representing 87.83% of the overall variance. In the PCA figure, solid circles of different colors denote separate clusters, while red arrows signify the direction and influence of each variable. Dashed circles highlight the dispersion range of each cluster, centered on mean values and extending to indicate standard deviation radii.

Cluster 1 samples are positioned in the upper right quadrant of the PCA plot, characterized by higher levels of nicotine and HA + FA and lower ratios of *Penicillium chrysogenum* H/C and *Bacillus subtilis* OD_600_. This configuration suggests an inhibitory effect on microbial growth. The longer arrows for nicotine and HA + FA point positively, indicating substantial contributions to PC1. Cluster 2 samples are scattered predominantly across the middle region of PC1 and PC2, with moderate levels of nicotine and HA + FA and slightly elevated ratios of *Penicillium chrysogenum* H/C and *Bacillus subtilis* OD_600_. This pattern suggests a less pronounced inhibitory effect. The arrows for *Penicillium chrysogenum* H/C and *Bacillus subtilis* OD_600_ point in the negative direction, signifying significant negative contributions to PC1. Cluster 3 samples are in the lower left quadrant, characterized by the lowest Nicotine content and highest ratios of *Penicillium chrysogenum* H/C and *Bacillus subtilis* OD_600_. This indicates a significant promoting effect on microbial growth.

PC1, which accounts for 66.25% of the variance, is predominantly influenced positively by Nicotine (0.475) and HA + FA (0.367), while *Penicillium chrysogenum* H/C (−0.589) and *Bacillus subtilis* OD_600_ ratios (−0.541) contribute negatively. PC2 explains 21.58% of the variance and is primarily driven positively by Nicotine (0.473) and negatively by HA + FA (−0.839).

**Figure 2:**
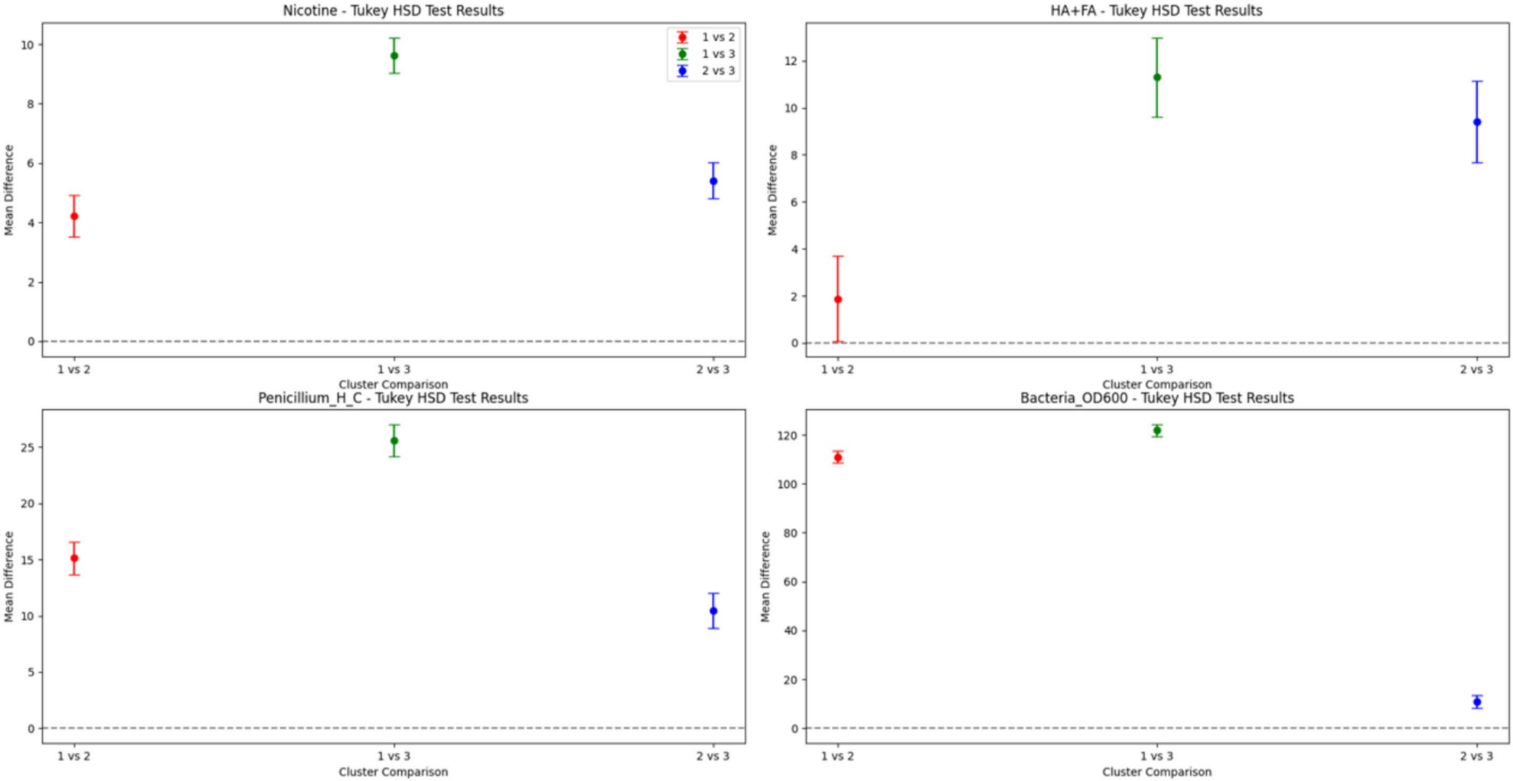
Turkey HSD test results for four components across categories.

Figure 2 presents the mean differences and confidence intervals for four characteristic components (nicotine, HA + FA, *Penicillium chrysogenum* H/C, and *Bacillus subtilis* OD_600_) across clusters (Cluster 1, Cluster 2, Cluster 3). Significant differences were observed for nicotine in all comparisons, with the largest difference between Cluster 1 and 3 (mean difference of 9.627). HA + FA also exhibited significant differences in all comparisons, notably between Cluster 1 and 3 (mean difference of 11.29). Both *Penicillium chrysogenum* H/C and *Bacillus subtilis* OD_600_ showed significant differences across all groups, with *Bacillus subtilis* OD_600_ demonstrating the most substantial difference between Cluster 1 and Cluster 3 (mean difference of 121.9). The findings indicate notable distribution variations of each component across the clusters, affirming the clustering results’ validity. They provide solid statistical evidence that supports additional research and data interpretation, highlighting the statistically significant differences in each component across categories.

In summary, nicotine and HA + FA play significant roles in influencing microbial growth. Cluster 1, characterized by high Nicotine content, exhibits lower microbial growth attributed to nicotine’s bacteriostatic effect (Yuan W. et al., 2022). Cluster 3, characterized by high HA + FA content, exhibits increased microbial growth, indicating that humic acids enhance microbial reproduction and activity. The findings highlight nicotine and HA + FA as significant indicators of microbial community distribution and development, underscoring their essential roles in the composting microbial ecosystem.

### 3.3 Hyperspectral images of HTS

#### 3.3.1 HSI spectra of HTS

**Figure 3:**
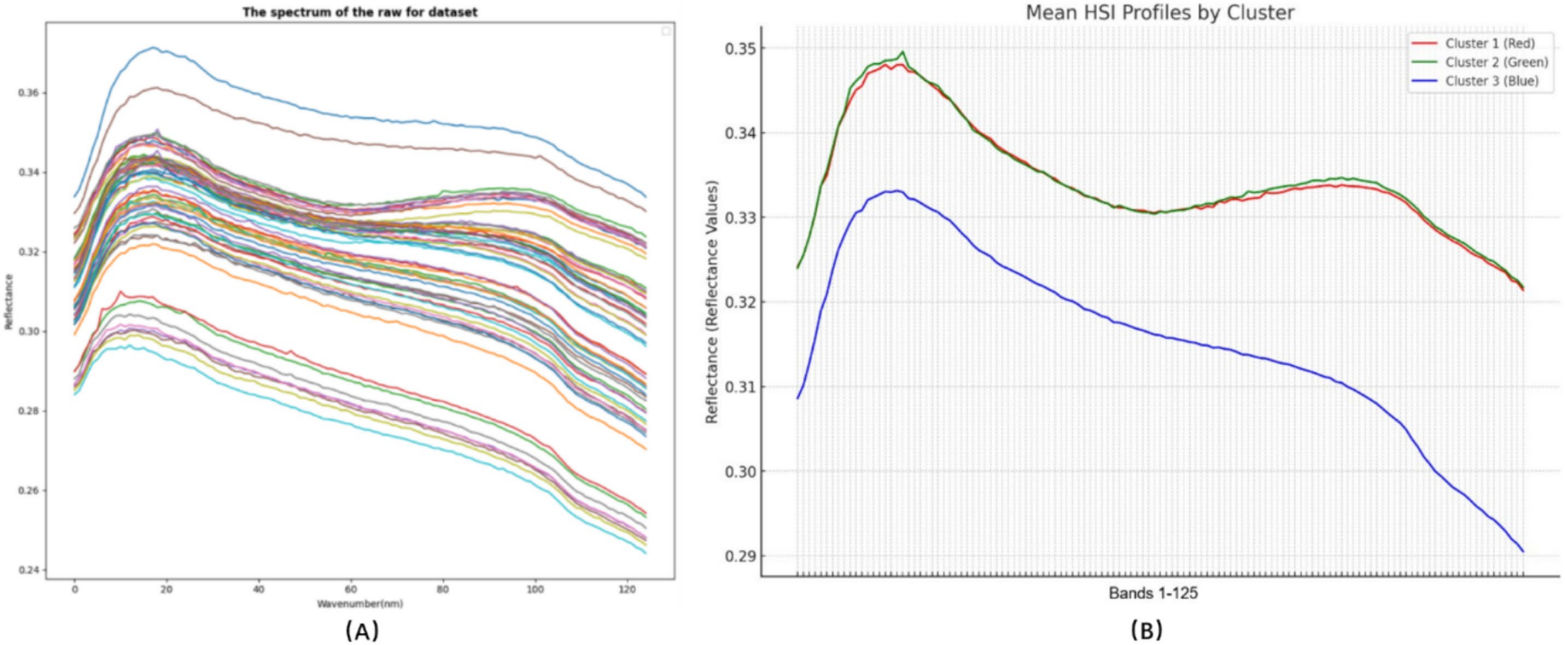
Hyperspectral imaging (HSI) profiles of HTS in the 900–1700 nm range. **(A)** HSI profiles of all hydrothermal products; **(B)** HSI profiles of classified hydrothermal products.

The HSI spectra of HTS exhibit a consistent trend within the wavelength range of 900–1700 nm. Significant differences in reflectance are evident among products subjected to different treatment intensities, with values ranging from 0.244 to 0.371 (Figure 3A). By averaging the spectral data and applying hierarchical cluster analysis (HCA), the hydrothermal products were classified into three categories (Figure 3B). The overall sequence of HSI spectral intensity is Cluster 1 ≈ Cluster 2 > Cluster 3, reflecting the properties of the hydrothermal products. HSI data correlate with hydrothermal products’ chemical and biological characteristics, indicating their potential utility in developing predictive models. The dataset comprising 125 wavelengths per HSI curve may present challenges, including increased computational complexity and noise in predictive modeling.

#### 3.3.2 Selection of characteristic wavelengths

HSI frequently encompasses tightly correlated adjacent bands, resulting in multicollinearity challenges among proximate wavelength variables. Feature wavelengths, essential for differentiating categories or identifying specific substances, generally display substantial alterations in the spectrum. According to previous hierarchical clustering studies, these feature wavelengths can be identified by examining average reflectance fluctuations across designated wavelengths for each group. This approach reduces data dimensionality, conserves storage space, and retains critical information, thereby mitigating multicollinearity and bolstering model robustness. This method improves model accuracy and generalization by reducing the utilized wavelengths. Four separate machine learning models were used to assess the Competitive Adaptive Reweighted Sampling (CARS) strategy for feature wavelength selection: Support Vector Machine (SVM), K-Nearest Neighbors (KNN), Random Forest (RF), and Extreme Gradient Boosting (XGBoost). These models were selected for their unique capabilities in managing high-dimensional data and non-linear connections. For each model, hyperparameter tuning was conducted using grid search and cross-validation to ensure optimal performance. The grid search method explored a range of parameter values for each model, and k-fold cross-validation (*k* = 10) was applied to avoid overfitting and to select the most appropriate combination of hyperparameters for each model.

For the SVM model, the optimal regularization parameter (C) and kernel type (linear or RBF) were selected based on their ability to maximize the calibration correlation coefficient (rc) while minimizing RMSE. In the KNN model, the number of neighbors (k) was adjusted, and the optimal value was selected by minimizing the prediction error (RMSEP). The number of decision trees (n_estimators) and the maximum tree depth (max_depth) were optimized for the RF model to balance model complexity and prediction accuracy. The XGBoost model required the adjustment of the learning rate (eta), the number of boosting rounds, and the maximum tree depth to enhance model accuracy and computational efficiency. This assessment evaluates the effectiveness of CARS in dimensionality reduction and in addressing multicollinearity issues, presenting performance results across various models. Table 3 presents a comparison of the performance of these models in selecting characteristic wavelengths.

**Table 3:** Performance comparison of selected characteristic wavelengths by different models.

Model	r_c_	r_P_	R_c_	R_p_	RMSEC	RMSEP
SVM	0.839	0.944	0.704	0.891	12.12	12.42
KNN	0.889	0.966	0.793	0.932	7.931	4.934
RF	0.979	0.966	0.959	0.933	3.584	4.849
XGBoost	0.999	0.935	0.997	0.874	0.941	6.579

Support Vector Machine (SVM), K Nearest Neighbors (KNN), Random Forest (RF) and Extreme Gradient Boost (XGBoost), correlation coefficients of calibration (r_c_), correlation coefficients of prediction (r_p_), coefficients of determination of calibration (R_c_), coefficients of determination of prediction (R_p_), root mean square error of calibration (RMSEC), and root mean square error of prediction (RMSEP).

Overall, the RF model demonstrates superior performance across various metrics, including calibration correlation values and calibration and prediction RMSE, highlighting its robust predictive capability on this dataset. The XGBoost model also shows strong performance in calibration correlation, albeit slightly trailing behind random forests in prediction correlation and RMSE. Conversely, the KNN and SVM models exhibit comparatively lower calibration and prediction performance, notably with higher RMSE errors. Consequently, the Random Forest model emerges as the optimal choice for hyperspectral data analysis due to its superior performance and suitability for this dataset.

**Figure 4:**
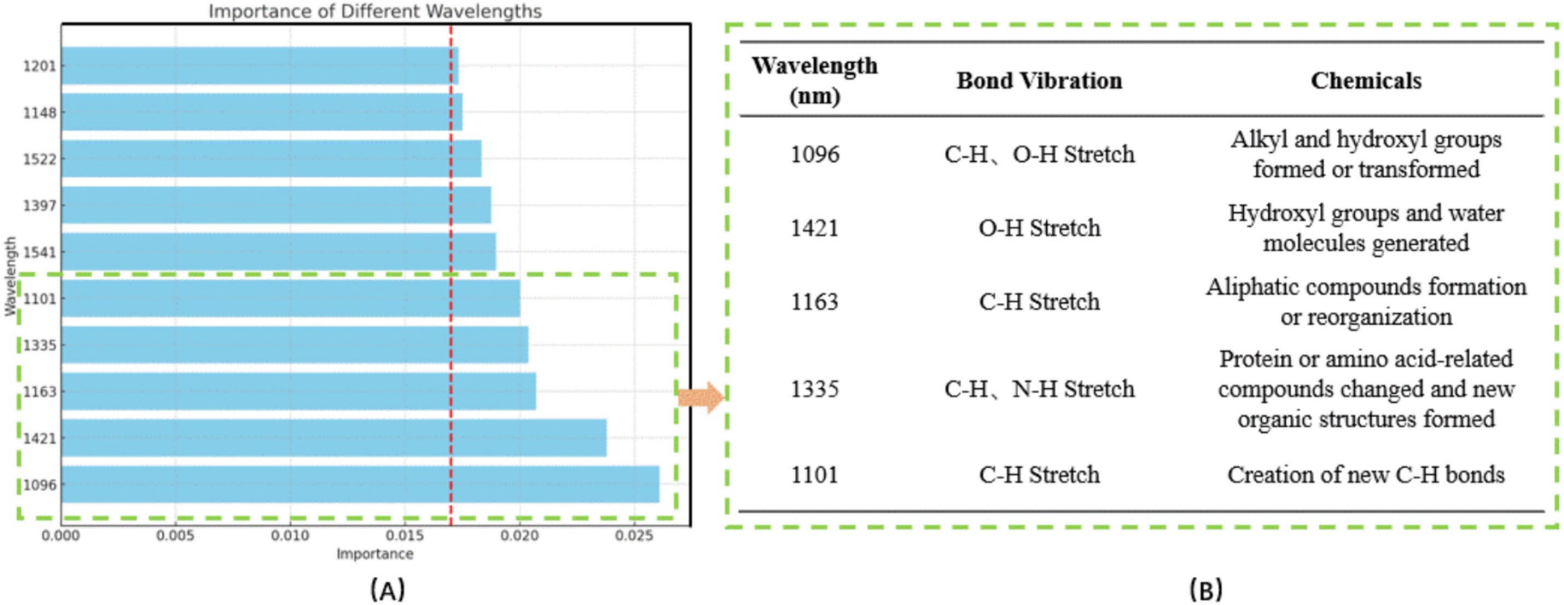
**(A)** Ten characteristic wavelengths obtained by RF and their importance; **(B)** Top five wavelengths and corresponding chemical bonds.

We utilized the CARS feature wavelength selection approach to improve model performance by minimizing complexity and identifying essential spectral bands to forecast hydrothermal products’ effects on microbial growth. This method identified wavelengths in the RF model with relevance scores beyond a predetermined threshold (e.g., 0.017). The importance score of each wavelength reflects its contribution to model accuracy, typically assessed by reducing impurity or errors in decision trees. Figure 4 illustrates the 10 selected feature wavelengths in the RF model and their corresponding chemical bonds. This method demonstrates the efficacy of integrating the CARS screening technique with RF modeling to attain superior outcomes in forecasting the impact of hydrothermal products on microbial proliferation.

The features identified through the CARS method accurately represent the chemical composition of tobacco stalks and their changes during hydrothermal processing. Tobacco stalks are composed mainly of cellulose, hemicellulose, and lignin. These components experience significant degradation and recombination reactions under elevated temperature and pressure hydrothermal conditions, forming new chemical bonds and molecular structures.

Figure 4B highlights the top five characteristic wavelengths and their corresponding chemical bonds. At 1096 nm and 1,101 nm, the stretching vibrations of C-H and O-H bonds predominate, which is characteristic of cellulose and hemicellulose degradation products, such as small sugars and alcohols. The wavelength of 1,421 nm is primarily associated with O-H bond stretching vibrations, signifying substantial generation of hydroxyl groups and reorganization of water molecules during hydrothermal treatment, leading to considerable absorption at this wavelength. The stretching vibrations of C-H and N-H bonds are apparent at 1163 nm and 1,335 nm, indicating partial breakdown and recombination of lignin into novel aliphatic and nitrogenous molecules. These newly formed chemical structures exhibit distinctive absorption features at these wavelengths.

The hydrothermal treatment of tobacco stalks involves complex chemical processes, encompassing dehydration, hydrolysis, condensation, and rearrangement reactions. These reactions lead to alterations in existing chemical bonds or the formation of new chemical bonds within the sample, resulting in distinct spectral absorption peaks at specific wavelengths (Xu et al., 2023; Provenzano et al., 2018). Nicotine, a prominent tobacco waste alkaloid, has a unique chemical structure. Its absorption characteristics in the spectrum differ notably from those of lignin and other aromatic compounds. Nicotine’s characteristic absorption occurs primarily in the ultraviolet region (200–300 nm) rather than in the visible light spectrum. The characteristic wavelengths mainly indicate alterations in polysaccharides, lignin, hemicellulose, and other constituents of the hydrothermal products derived from tobacco stalks.

### 3.4 Predicting the impact of HTS on composting microbial growth using HSI and RF model

#### 3.4.1 RF model prediction

**Figure 5:**
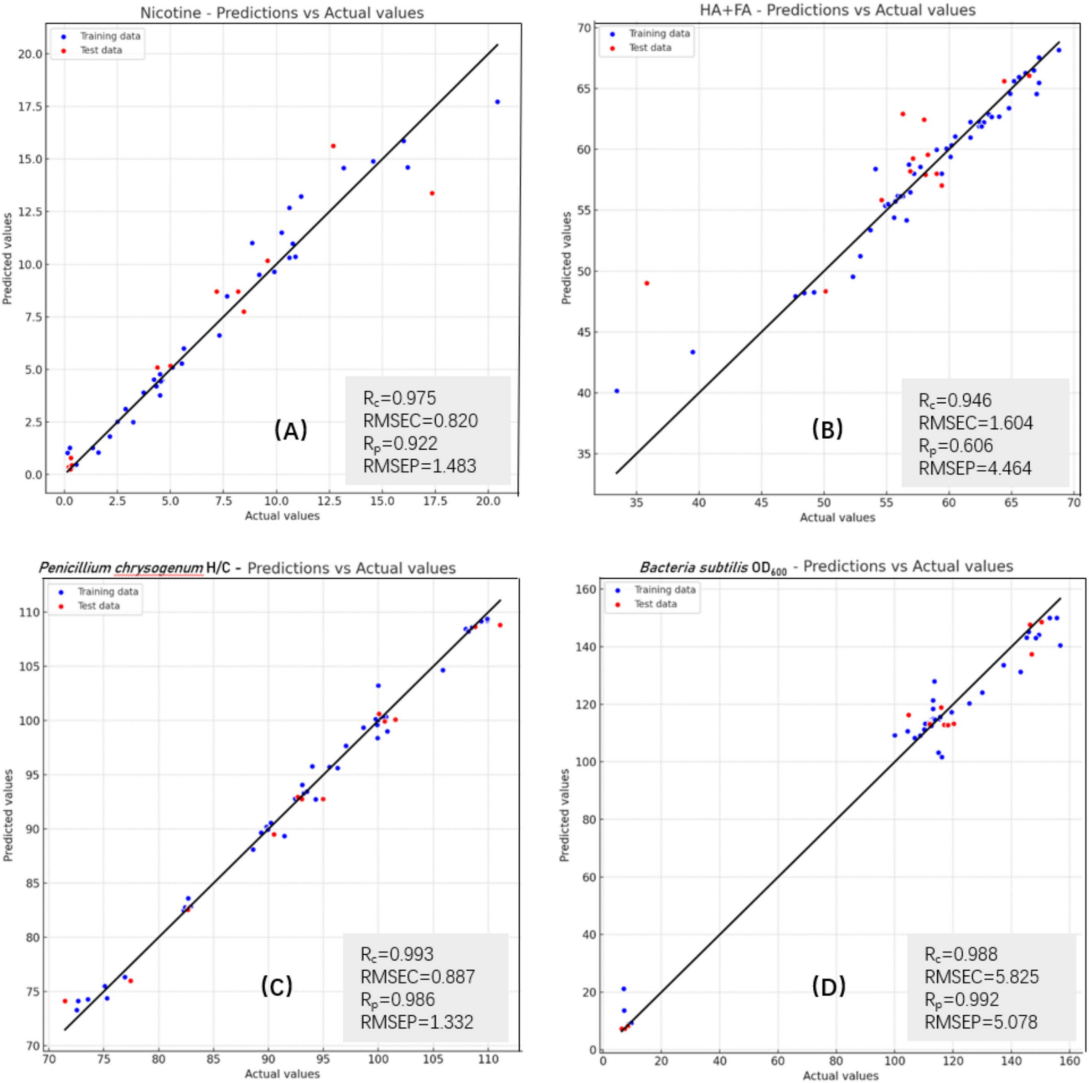
RF model prediction curves and model characteristics for four components: **(A)** Nicotine content; **(B)** HA + FA content; **(C)** *Penicillium chrysogenum* H/C ratio; **(D)** *Bacillus subtilis* OD_600_ ratio.

The RF model was utilized to forecast four key components: nicotine, humic acid + fulvic acid (HA + FA), *Penicillium chrysogenum* H/C ratio, and *Bacillus subtilis* OD_600_ ratio. Figure 5 depicts the performance of the RF model in forecasting these components, together with the associated model performance measures. The RF model demonstrated significant accuracy in predicting nicotine and HA + FA levels, with prediction curves nearly matching actual data, thereby confirming the effectiveness of the selected wavelength characteristics. Predicting HA + FA content exhibited a lower R_P_ value (0.606) and higher RMSE (4.464), suggesting greater variability in HA + FA content across samples or potential model limitations in capturing this feature’s complexity. In contrast, predictions for *Penicillium chrysogenum* H/C and *Bacillus subtilis* OD_600_ ratio proved more precise, achieving R_C_ and R_P_ values exceeding 98.5%. This demonstrates the model’s ability to accurately represent microbial strain growth trends across different treatment conditions, emphasizing its efficacy in quantitative microbial analysis and practical applications.

The performance metrics for all four characteristic components demonstrate significant accuracy and consistency. The RMSE values in the prediction set are slightly elevated compared to the calibration set, suggesting a minor increase in prediction error for new data; nonetheless, the total errors remain within acceptable thresholds. Consequently, this methodology enhances analytical accuracy and efficacy, providing a dependable instrument for forthcoming studies, especially in environmental microbiology and the optimal utilization of agricultural waste resources.

#### 3.4.2 Confusion matrix of the RF model

**Figure 6:**
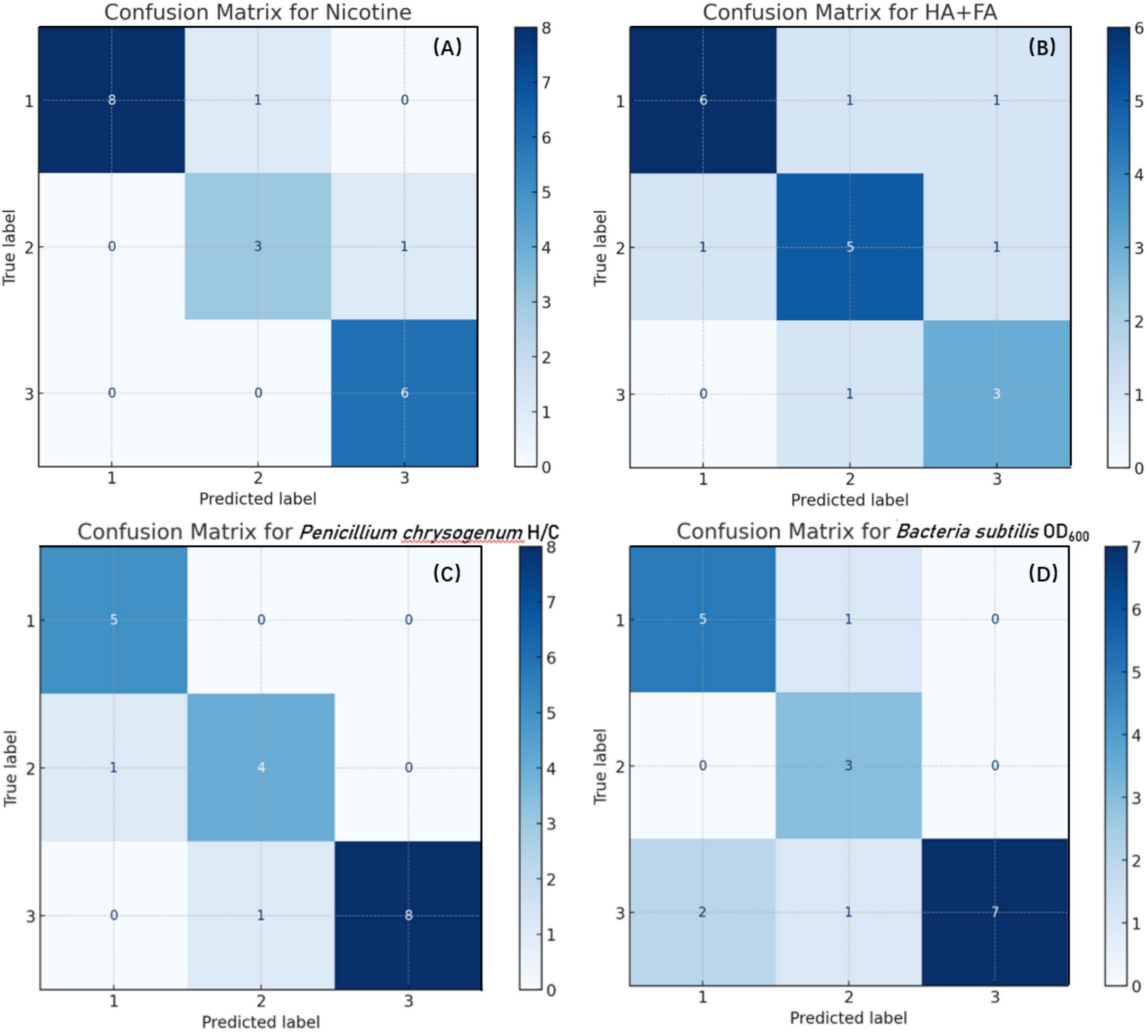
Confusion matrices of the RF model for classification based on four feature components, with numbers 1, 2, and 3 on the axes corresponding to Cluster 1, 2, and 3, respectively: **(A)** Nicotine content; **(B)** HA + FA content; **(C)** *Penicillium chrysogenum* H/C ratio; **(D)** *Bacillus subtilis* OD_600_ ratio.

Images of hydrothermal products were entered into the model, and the accuracy of the category predictions was evaluated. The evaluation measured performance using a confusion matrix by comparing actual and expected categories. Figure 6 illustrates the confusion matrix of the RF model for predictions of the four types of feature components, where axes labeled 1, 2, and 3 correspond to Cluster 1, 2, and 3, respectively. Off-diagonal elements display low non-zero values, suggesting a low misclassification rate, while diagonal elements reveal correct predictions for each class. Owing to the small sample size, there are 42 samples in the training set and 19 in the test set. To ensure that the model can adequately learn during training and effectively evaluate performance during testing, the proportion of samples assigned to the training and test sets is deemed suitable.

Most test set samples were identified correctly, indicating the excellent accuracy of the confusion matrix for predicting nicotine levels. This demonstrates how well spectral data acquired using HSI technology may represent changes in nicotine concentration under various hydrothermal settings. It confirms the sensitivity and logic of feature wavelength selection and offers a solid basis for future studies into modifications in tobacco chemical composition. HSI technology acquires spectral data across multiple wavelengths, facilitating the investigation of chemical composition and variations. The RF model handles high-dimensional data and identifies essential wavelengths among various features, thus attaining substantial classification accuracy.

The RF model successfully follows changes in humic and fulvic acid content throughout hydrothermal treatment, as evidenced by the predictions for these components’ contents. It achieves good accuracy with a slightly raised misclassification rate. This is consistent with the performance and prediction curve of the RF model shown in Figure 5B. The complexity of spectral characteristics for humic acid and fulvic acid, alongside subtle changes under varying hydrothermal conditions, may contribute to these challenges in category differentiation (Rodríguez et al., 2014). However, the overall prediction findings highlight the RF model’s strong performance and guide further improvements and optimizations in subsequent studies.

The RF model succeeds in predicting the ratios of *Penicillium chrysogenum* H/C and *Bacillus subtilis* OD_600_, with elevated prediction accuracy and a minimal misclassification rate. This highlights the model’s capacity to accurately capture the dynamics of colony ratios under various treatment settings, thus validating its significant utility in evaluating microbial community structures.

## 4 Discussion

In recent years, integrating the RF model and HSI has significantly advanced prediction and classification tasks across various areas. This methodology has shown significant results in agriculture, environmental science, and food quality evaluation and shows potential in chemical composition analysis. Wu et al. (2020) utilized HSI data with the RF model to precisely classify wheat diseases, significantly enhancing disease recognition efficiency. Xu et al. (2021) successfully detected and classified soil contamination swiftly using HSI data and RF, offering an efficient tool for environmental monitoring. Lan et al. (2021) showcased rapid detection of sugar content and acidity in apples using HSI and RF, validating its effectiveness in food quality assessment. Divyanth et al. (2022) achieved accurate classification and quantitative analysis of diverse chemical components in tobacco using HSI data paired with the RF model. In conjunction with HSI, the RF model has exhibited significant benefits and a wide range of potential applications in various fields. To further improve the accuracy and reliability of predictions, future research can concentrate on refining data preprocessing techniques and optimizing model parameters, thereby expanding the application potential.

The method proposed in this study for predicting compostability in hydrothermally treated tobacco straw using hyperspectral imaging (HSI) and chemometric modeling offers a rapid and efficient assessment tool. This approach streamlines the analysis process by leveraging HSI data and hydrothermal conditions, reducing the complexity and time required compared to traditional chemical and microbiological methods. However, the model’s reliability is restricted by the current dataset of 61 samples, as larger datasets typically enhance the model’s performance. In this context, non-linear approaches, such as Random Forest, which employs tree regression methods, are more appropriate than linear models, which frequently confront challenges with non-linear spectral effects.

The four models developed in this study were calibrated and validated using our experimental data, which specifically evaluated the impact of hydrothermal products on composting microorganisms. Since no models are specifically designed for composting with hydrothermally treated tobacco straw, external datasets were not used for validation. Despite having 61 experimental groups, the study’s data trustworthiness was ensured by the assurance that each group was based on actual hydrothermal processing. Similar studies’ models might not directly apply to this work due to significant changes in experimental methods and materials We acknowledge that there are only a few experimental groups; future studies will concentrate on increasing the dataset by including more varied tobacco waste samples, differing in kind, source, processing amount, and composting conditions. This will enable more validation and enhancement of the dependability and applicability of the model.

Simple color-based image thresholding yielded favorable outcomes in extracting regions of interest (ROI) and calculating average spectra in this study. Future research should explore more complex ROI segmentation techniques, including deep learning approaches and clustering algorithms, in light of the tobacco industry’s diverse conditions. These developments will improve the reliability and applicability of the proposed method in real-world contexts.

## 5 Conclusion

The results of this study confirm that a promising substitute for conventional chemical analysis in assessing the compostability of tobacco waste that has undergone hydrothermal processing is the combination of HSI and ML algorithms. This approach provides a rapid and convenient method, particularly effective in predicting nicotine content, total humic acid content, and their influence on the growth of composting microorganisms like *Penicillium chrysogenum* and *Bacillus subtilis*. Samples were divided into three groups using HCA and PCA according to the effects of heat treatment on composting: augmentation, inhibition, and no effect. Using CARS, the study assessed four machine-learning methods (SVM, KNN, XGBoost, and RF) to determine which spectral bands were most important for modeling. Except for total humic acid concentration (60%) and four important variables, RF showed good predictive potential, obtaining satisfactory findings with prediction accuracies exceeding 90%.

This study establishes the groundwork for understanding how hyperspectral images correlate with the chemical composition and compostability of hydrothermally processed tobacco straw products. The feasibility of predicting tobacco straw hydrothermal composting outcomes using hyperspectral images has been demonstrated. However, additional enhancements are required for machine learning predictive models. Subsequent research will prioritize increasing sample numbers and including more physicochemical indicators to improve the model’s overall efficacy. This will precisely assess composting success rates based on diverse physicochemical properties of hydrothermally processed materials.

## Data Availability

The original contributions presented in the study are included in the article/Supplementary material, further inquiries can be directed to the corresponding author.
