# Supplementary material for: Prediction of the impact of tobacco waste hydrothermal products on compost microbial growth using hyperspectral imaging combined with machine learning
Source: Front Microbiol. 2024 Nov 5;15:1476803. doi: 10.3389/fmicb.2024.1476803 (PMC11573759; doi:10.3389/fmicb.2024.1476803)
Supplement: Supplementary file 1 [file Table_1.DOCX]

**Supplementary Materials**

**Table S1. Basic Physicochemical Characteristics of Tobacco Straws**

| Item | pH | Moisture  (%) | EC  (mS/cm) | Starch  (%) | Protein  (%) | Cellulose  (%) | Hemicellulose  (%) | Lignin  (%) | Ash  (%) |
| --- | --- | --- | --- | --- | --- | --- | --- | --- | --- |
| Content | 5.3± 0.1 | 8.5 ± 0.2 | 1.1 ± 0.2 | 2.3 ± 0.3 | 4.8 ± 0.3 | 37.5 ± 1.1 | 22.7 ± 0.9 | 21.2 ± 0.8 | 9.6 ± 0.4 |

**Table S2.** **Hydrothermal treatment and Index content for HTS**

| **Group** | **Temp.**  **(°C)** | **Time**  **(min)** | **Nicotine**  **(%)** | **HA+FA**  **(mg/g)** | ***Penicillium chrysogenum* H/C ratio** | ***Bacillus subtilis* OD_600_ ratio.** |
| --- | --- | --- | --- | --- | --- | --- |
| 1 | 100 | 20 | 0.18 | 35.8 | 111.09 | 104.71 |
| 2 | 100 | 40 | 0.19 | 39.5 | 109.92 | 104.38 |
| 3 | 100 | 80 | 0.54 | 47.7 | 109.93 | 110.47 |
| 4 | 100 | 90 | 0.57 | 48.4 | 108.54 | 113.22 |
| 5 | 100 | 100 | 0.38 | 49.2 | 109.37 | 115.15 |
| 6 | 100 | 110 | 0.26 | 50.1 | 108.82 | 115.94 |
| 7 | 100 | 140 | 0.19 | 52.3 | 107.95 | 125.56 |
| 8 | 100 | 150 | 0.22 | 52.9 | 108.21 | 129.92 |
| 9 | 100 | 170 | 0.2 | 53.7 | 105.87 | 137.22 |
| 10 | 150 | 30 | 0.24 | 54.1 | 99.94 | 143.09 |
| 11 | 150 | 60 | 0.25 | 55.6 | 99.73 | 145.18 |
| 12 | 150 | 80 | 0.26 | 56.8 | 99.94 | 145.82 |
| 13 | 150 | 90 | 0.28 | 57.1 | 100.06 | 146.3 |
| 14 | 150 | 100 | 0.3 | 56.3 | 100.58 | 146.93 |
| 15 | 150 | 110 | 0.34 | 57.2 | 100.44 | 148.27 |
| 16 | 150 | 120 | 0.39 | 56.6 | 100.27 | 149.36 |
| 17 | 150 | 140 | 0.34 | 58 | 101.55 | 150.29 |
| 18 | 150 | 150 | 0.26 | 62.4 | 99.94 | 153.07 |
| 19 | 150 | 170 | 0.19 | 63.4 | 100.69 | 155.52 |
| 20 | 150 | 180 | 0.13 | 67 | 100.82 | 156.69 |
| 21 | 180 | 30 | 1.32 | 67.2 | 99.89 | 113.11 |
| 22 | 180 | 60 | 1.59 | 64.8 | 98.64 | 113.54 |
| 23 | 180 | 80 | 2.13 | 64.9 | 97.05 | 113.09 |
| 24 | 180 | 90 | 2.88 | 64 | 96.28 | 113.32 |
| 25 | 180 | 100 | 2.49 | 63.2 | 95.54 | 112.84 |
| 26 | 180 | 110 | 3.23 | 62.4 | 93.99 | 112.67 |
| 27 | 180 | 120 | 3.73 | 62.8 | 92.39 | 112.53 |
| 28 | 180 | 140 | 4.5 | 61.7 | 93.06 | 111.18 |
| 29 | 180 | 150 | 4.33 | 60.5 | 92.41 | 110.05 |
| 30 | 180 | 170 | 4.54 | 60.2 | 93.5 | 108.64 |
| 31 | 180 | 180 | 4.59 | 59 | 93.21 | 106.8 |
| 32 | 200 | 30 | 7.18 | 59 | 94.97 | 120.18 |
| 33 | 200 | 60 | 7.66 | 59.4 | 94.29 | 119.54 |
| 34 | 200 | 80 | 8.19 | 58.1 | 93.03 | 118.24 |
| 35 | 200 | 90 | 8.46 | 56.9 | 92.66 | 116.97 |
| 36 | 200 | 100 | 8.85 | 56.3 | 91.45 | 116.13 |
| 37 | 200 | 110 | 9.9 | 56 | 89.81 | 115.57 |
| 38 | 200 | 120 | 16.2 | 55.7 | 88.59 | 114.99 |
| 39 | 200 | 140 | 10.89 | 56.1 | 89.32 | 113.85 |
| 40 | 200 | 150 | 10.61 | 55.2 | 89.94 | 113.16 |
| 41 | 200 | 170 | 9.58 | 54.6 | 90.49 | 111.91 |
| 42 | 200 | 180 | 9.19 | 54.9 | 90.22 | 111.08 |
| 43 | 220 | 30 | 10.24 | 55.1 | 82.39 | 7.25 |
| 44 | 220 | 60 | 10.61 | 55.9 | 82.34 | 7.39 |
| 45 | 220 | 90 | 10.77 | 56.9 | 82.68 | 7.06 |
| 46 | 220 | 100 | 11.17 | 57.7 | 82.27 | 7.19 |
| 47 | 220 | 110 | 12.68 | 58.3 | 82.71 | 7.22 |
| 48 | 220 | 120 | 16.01 | 59.8 | 82.88 | 7.17 |
| 49 | 220 | 140 | 20.43 | 60.1 | 82.95 | 7.31 |
| 50 | 220 | 150 | 17.34 | 59.4 | 82.66 | 7.46 |
| 51 | 220 | 170 | 14.56 | 61.7 | 82.69 | 7.59 |
| 52 | 220 | 180 | 13.17 | 62.6 | 82.88 | 8.19 |
| 53 | 260 | 60 | 7.31 | 68.8 | 72.55 | 9.64 |
| 54 | 260 | 100 | 5.62 | 67.2 | 75.29 | 8.75 |
| 55 | 260 | 110 | 5.53 | 66.8 | 76.92 | 8.59 |
| 56 | 260 | 120 | 5 | 66.4 | 77.45 | 8.51 |
| 57 | 260 | 140 | 4.5 | 65.6 | 75.09 | 7.91 |
| 58 | 260 | 150 | 4.21 | 66.1 | 73.54 | 7.58 |
| 59 | 260 | 170 | 5.08 | 65.2 | 72.66 | 6.62 |
| 60 | 260 | 180 | 4.37 | 64.4 | 71.47 | 6.42 |
| 61 | 0 | 0 | 0.19 | 33.4 | 100 | 100 |
